# Evaluation of the behaviour change communication and community mobilization activities in Myanmar artemisinin resistance containment zones

**DOI:** 10.1186/s12936-015-1047-y

**Published:** 2015-12-23

**Authors:** Myat Htut Nyunt, Khin Myo Aye, Myat Phone Kyaw, Khin Thet Wai, Tin Oo, Aye Than, Htet Wai Oo, Hnin Phyu Phway, Soe Soe Han, Thurein Htun, Kyaw Kyaw San

**Affiliations:** Department of Medical Research, Yangon, Republic of the Union of Myanmar

**Keywords:** Malaria, Behaviour change communication, Community mobilization, Myanmar

## Abstract

**Background:**

Behaviour change communication (BCC) can improve malaria prevention and treatment behaviour. As a one of the activities under Myanmar Artemisinin Resistance Containment (MARC) programme, BCC have been conducting. This study aimed to evaluate the effectiveness of the behaviour change communication and community mobilization activities in MARC zones in Myanmar.

**Methods:**

A cross sectional descriptive survey was conducted in randomly selected 16 townships in Tier I and II areas of MARC zones by quantitative and qualitative approaches.

**Results:**

In 832 households resided by 4664 people, there were 3797 bed nets. Around 54 % were untreated while 45.6 % were insecticide-treated nets (ITN) and 36.2 % were long-lasting insecticide-treated nets (LLINs). Proportion of households with at least one ITN was 625 (75.12 %), proportion of households with at least one ITN for every two peoples was 487 (58.53 %), and proportion of existing ITNs used in previous night was 1225 (70.65 %) respectively. Nearly 23 % of households had old nets while 52 % had new and unused extra bed nets reflecting the adequacy. Interestingly, 38 % could not mention the benefit of the use of ITN/LLINs. Although 88.2 % knew the disease “malaria”, 11.9 % could not be able to mention the symptoms. More than 80 % provided correct responses that mosquito bite can cause malaria while only 36.9 % could mention the blood test for malaria diagnosis. Only 36.6 % received malaria information within previous year but nearly 15 % could not recognize it. Mostly, 80 % of fever episodes were treated at rural health centers (38.24 %) followed by drug shops (17.65 %) and private clinics (16.18 %) respectively.

**Conclusions:**

Efforts should focus on correcting misconceptions about malaria transmission, prevention and universal use of ITN/LLINs. Although BCC activities have been documented, it is still necessary to intensify community mobilization through all accessible multiple channels in MARC areas.

## Background

Artemisinin-based combination therapy (ACT) is recognized as the most effective pharmacological treatments of *Plasmodium falciparum* infection [1]. However, an reduced susceptibility of *P. falciparum* to artemisinin was reported at the Thai–Cambodian border in 2007 [2] and in southern Myanmar in 2011 [3, 4]. Therefore, a multifaceted containment programme has been launched, including early diagnosis and appropriate treatment, decreasing drug pressure, optimizing vector control, targeting the mobile population, strengthening management and surveillance systems, and operational research [5]. To guide implementation, malaria endemic countries should consider the following three classifications: Tier I areas for which there is credible evidence of artemisinin resistance; Tier II areas with significant inflows of people from tier I areas, including those immediately bordering tier I; Tier III areas with no evidence of artemisinin resistance and limited contact with tier I areas [4]. In Myanmar, the Myanmar Artemisinin Resistance Containment programme (MARC) has been conducted by the tier approach since 2011 [5].

The fifth objective of the MARC strategy mentions as “to support containment/elimination of artemisinin tolerant parasites through comprehensive behaviour change communication (BCC), community mobilization and advocacy” [5]. BCC is defined as any intervention with individuals or communities to develop communication strategies to promote positive behaviours appropriate to their settings to provide a supportive environment enabling people to initiate and sustain the desired behaviours, such as sleeping under insecticide-treated bed nets (ITN) [6]. There is evidence that effective BCC programmes can increase a range of positive health behaviours in a target population [7, 8]. BCC has a significant effect on utilization of ITN for prevention of malaria [9].

**Fig. 1:**
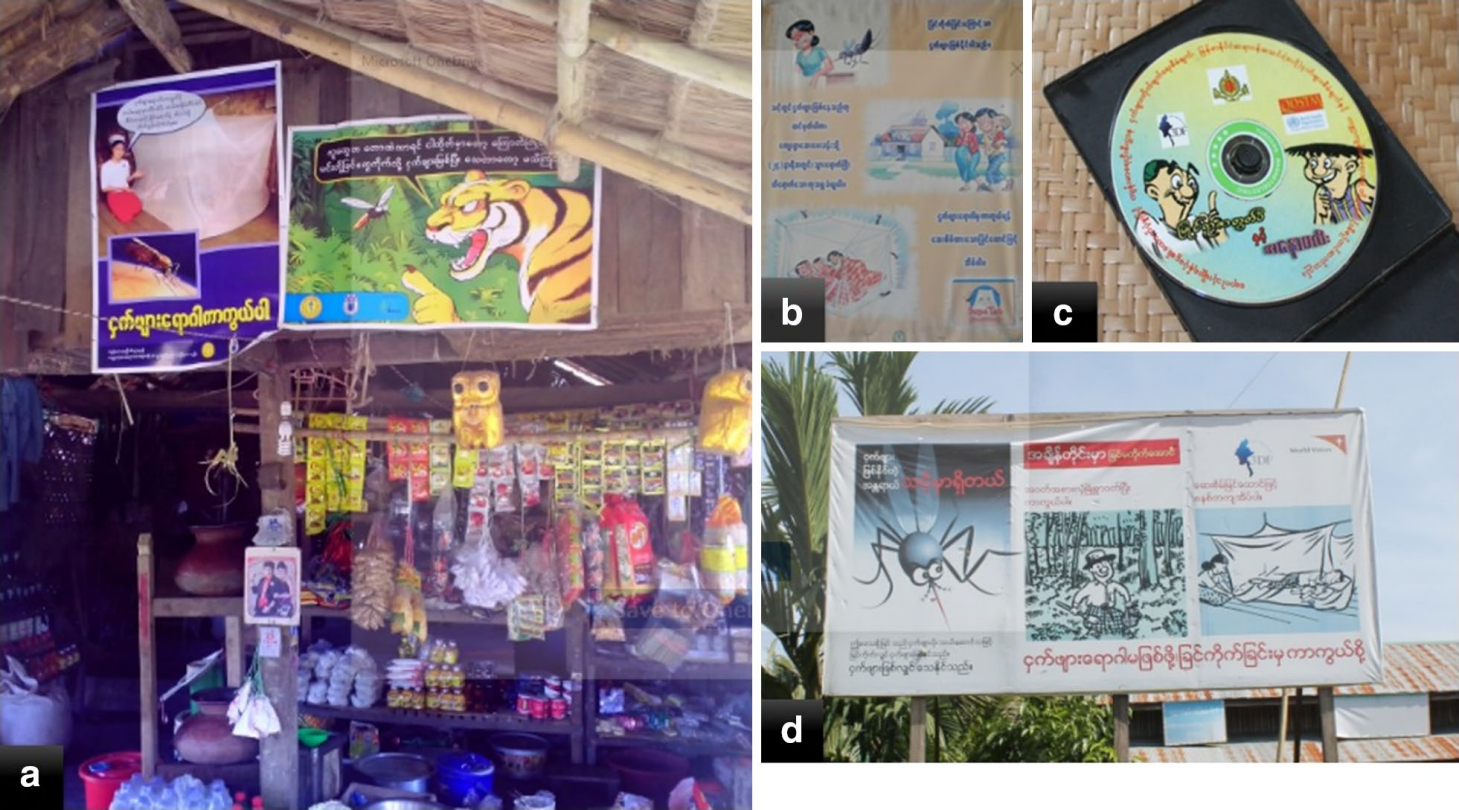
IEC materials distributed in study sites. Various IEC (information, education and communication) materials were distributed for BCC (behaviour change communication) in target population. **a** Posters applied in local grocery shop, **b** Pamphlet, **c** Video disk (VCD), **d** Billboard showing the cause of malaria and prevention methods

In Myanmar, regional artemisinin initiatives (RAI) have been implemented and it covered the drug resistance falciparum malaria among mobile and migrant population, distribution of ITN, investigation of all falciparum cases in low endemic areas, comprehensive response package to all falciparum cases, administration of the direct observed treatment, quality assurance and control of diagnosis and treatment of malaria. However, provision of the diagnosis, treatment and preventive tools are not enough to combat the disease without proper information to change their risk behaviour by conjunction with all interventions to increase the knowledge and practice of the target population. Therefore, mass media, community networks and interpersonal approached have been used to deliver the BCC activities in all target townships in Tier I and II areas of MARC. IEC materials, such as pamphlets, posters, bill boards, have been distributed (Fig. 1). All of the basic health staff and malaria volunteer were trained for interpersonal communication and health talk to promote the BCC in target population. Mass media including radio talk, television short stories and video have been developed after conducting the containment efforts [5, 6].

**Fig. 2:**
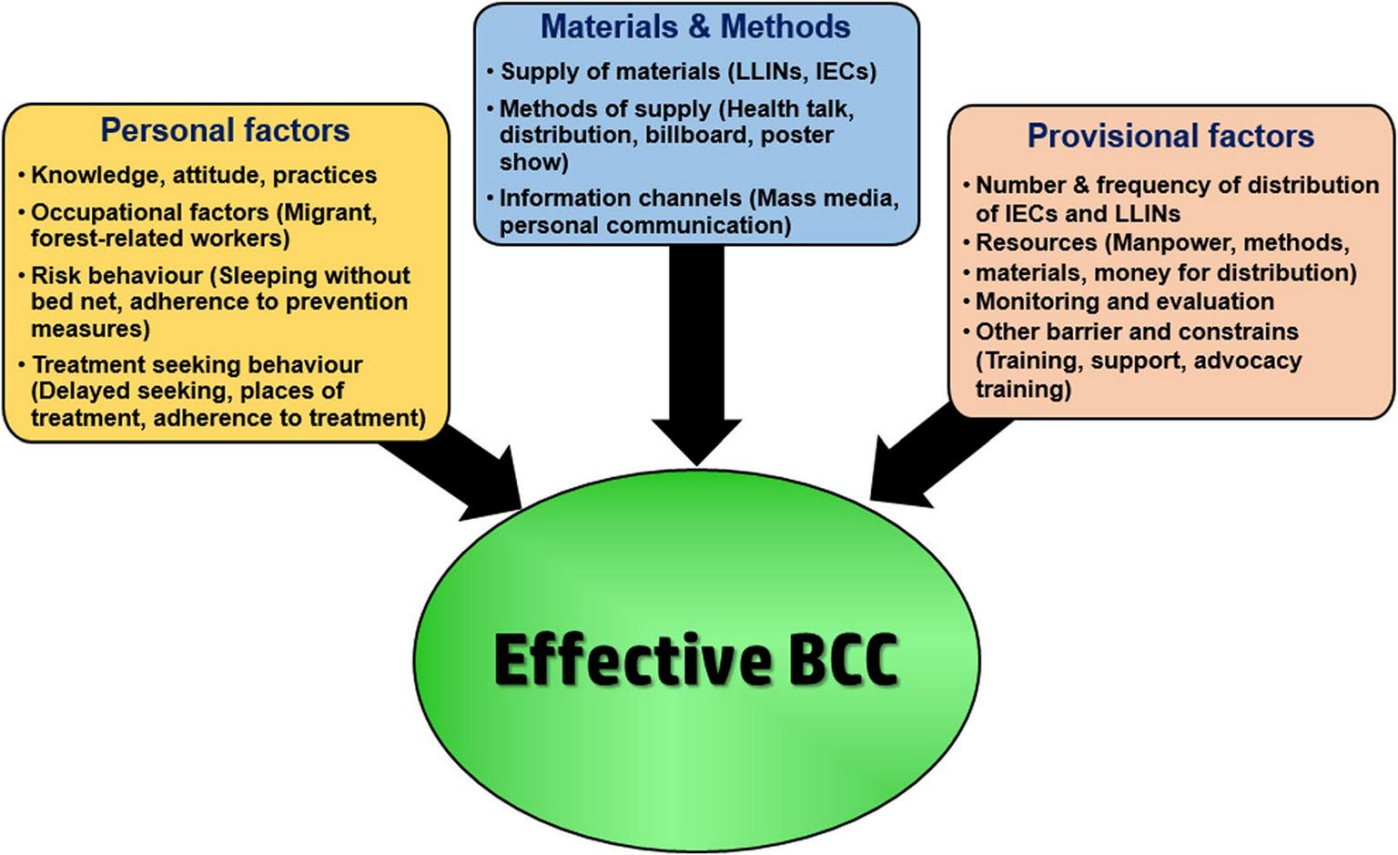
Conceptual frame work. Many factors of the communities, providers and materials will contribute the successfully achievement of behavior change communication (BCC). *IEC* information, education and communication, *LLIN* long lasting insecticidal nets)

Although BCC activities and community mobilization activities have been ongoing in MARC zones, there may be many possible factors and barriers to a successful programme (Fig. 2). In this study, the effectiveness of the BCC activities in target audience, Tier I and II of the Myanmar artemisinin resistance containment zones was assessed.

## Methods

### Study design and setting

The field based cross sectional descriptive study with analytical components was conducted from September to December, 2013. This study was carried out in selected 16 townships from the Myanmar artemisinin containment zones (Kayin and Mon State; and Bago and Thanintharyi Region). At time of survey, Bago (Shwe Kyin Township), Mon State and Thanintharyi Region was defined as Tier I and Kayin State and Bago (other townships) as Tier II. In 2014, all of the Tier II areas were recognized as Tier I [10].

### Sample size determination and sampling procedure

The study design is multi-stage, sampling clusters at the first stage, households within each cluster at the second stage, and then individuals within each household. Based on the population figures and natural proportions by risk category (Strata 1a, 1b, 1c, 2 and 3) in Tier 1 and 2, and overall malaria prevalence of 15 %, a sample size of 800 households provided a precision of ±3 % assuming a design effect of 1.75, non-response rate of 10 %, power of 80 % (beta error), and confidence interval of 95 % (alpha error). As most villages should have at least 25 households, it is likely that each selected cluster would consist of a single village [11].

All of the townships were listed and four townships were randomly selected in each of the study sites by adding the sampling interval i.e., total number of the townships divided by 4. Similarly, five villages from each townships and 10 households from each villages were also selected.

To find out the effectiveness of the BCC activities in targeted areas, community-based household surveys were conducted using qualitative and quantitative approaches. Pre-coded and pre-tested structured questionnaires were used for face-to-face interviews. The questionnaires were designed to collect information on household characteristics, family size, nearest health facilities, bed net ownership and utilization, knowledge about malaria and mosquito nets, source of information and treatment-seeking behaviour. Interviews were conducted by trained interviewers in the local languages using questionnaires adapted and modified from the Malaria Consortium [11].

Focus group discussions (FGD) were done at two places in each study sites by stratified purposive sampling method. Discussion guidelines were formulated as a series of open-ended questions. Key informal interviews were also conducted from local health authorities, community leader, township health officer and State/Region health officer.

### Data management

Data collected during the survey were checked and entered by using Microsoft Excel and SPSS version 22. Analysis was performed using SPSS (version 22.0, IBM SPSS Statistics, Armonk, New York, USA). Pearson’s Chi squared test was used to determine association with a *P* value of <0.05 accepted as significant. Logistic regression was calculated using the selected independent variables to estimate the outcome variables. Mann–Whitney test was used to for nonparametric analysis on non-normal distribution. Qualitative data was transcribed and analysed across themes and sub themes for triangulation with quantitative data.

### Ethical consideration

Participation in this project was entirely voluntary. This project was obtained ethical clearance from the ethical committee of the Department of Medical Research (Approval No-56/Ethics, 2013).

## Results

In this cross-sectional descriptive study, a total of 832 households resided by 4664 peoples were included from four townships each from Bago, Thanintharyi Region and Mon, Kayin State. More than half of the households (55.0 %) had the 4–6 family members while 26.8 % had 7–10 members in their household. Medium income was 120,000 Kyats per month and 78.1 % of them were irregular. Basic demographic characteristics of the study population were shown in Table 1.

**Table 1:** Basic demographic characteristics of the respondents

Category	Description	Number	Percent (n = 832)
Sex	Male	242	29.09
	Female	590	70.91
Education	Not attend	85	10.22
	Can read and write	65	7.81
	Primary	381	45.79
	Middle	170	20.43
	High school	89	10.70
	College or University	42	5.05
Ethnicity	Myanmar	430	51.70
	Kayin	287	34.50
	Mon	71	8.50
	Others	44	5.30
Family member	1–3 members	134	16.11
	4–6 members	458	55.05
	7–10 members	223	26.80
	>10 members	17	2.04
Family income per month	<50,000 MMK	88	10.58
	50,001–100,000 MMK	306	36.78
	100,001–200,000 MMK	330	39.66
	200,001–400,000 MMK	94	11.30
	>400,000 MMK	14	1.68
Situation of income	Regular	180	21.63
	Not regular	650	78.13
	Uncertain/not sure	2	0.24
Assets for media	Radio	435	52.28
	Television	492	59.13
	DVD player	467	56.13
	Mobile phone	230	27.64

Rural Health Centers (RHC) were selected as nearest health care provider by most of them (64.8 %). Although almost villages had the village health/malaria volunteer, only 81 (9.7 %) accepted them as first nearest health care persons while 26 (3.1 %) recognized the quacks, a person who dishonestly claims to have medical knowledge or skills, for their health care.

Among the 832 households resided by 4664 people, there were 3797 bed nets. Almost all families (99.8 %) had a bed net. Most of them had 3–5 bed nets in their family. Among the nets, 1734 (45.6 %) were ITN/LLINs, 1377 (36.2 %) were LLIN and 2063 (54.4 %) were untreated nets. More than 30 % of all bed nets were received within 1 year. For the untreated bed nets, majority of the source of nets were from shop/market while government and non-governmental organizations (NGOs) were main source of treated net providers (26 and 10 % of all nets respectively).

Almost all distributed LLINs were family size and only 9.4 % of the all bed net observed in this study were single size. Regardless of the types of the bed nets, 943 (24.8 %) were received re-treatment by insecticide. Most of the re-treatment activities were conducted in the village (785, 83.2 %) and mostly (890, 94.4 %) free of charge by health personals and NGOs. Interestingly, 2346 nets (61.8 %) were being used last night before survey while 1431 (37.7 %) were not being used. Proportion of existing ITNs used the previous night was 70.65 %.

There were 433 households (52.0 %) that had the new bed nets, while (191, 23 %) had old unused nets. They said that the new nets were being saved because they were adequate nets (102, 23.5 %), it was for visitors (238, 54.8 %) and for future use (56, 12.9 %). Among the participants, 320 (38.46 %) did not know the benefit of ITN/LLINs (Table 2).

**Table 2:** Knowledge, ownership and use of bed net

Category	Description	Number	Percent (%)
No. of bed net in their household	1–2	153	18.39
	3–5	545	65.50
	6–8	116	13.94
	>8	16	1.92
Type of bed net	ITN/LLIN	1734	45.67
	Untreated bed nets	2063	54.33
Damage of the net	Nets with holes	787	20.73
	Repaired nets	409	10.77
Retreatment	Retreated bed net	943	24.84
	Retreatment of ITN	432	11.37
Frequency of washing	Weekly	175	4.61
	Monthly	822	21.65
	Every 2–3 months	851	22.41
	Twice per year	526	13.85
	Once per year	312	8.22
	Less than once a year	147	3.87
	Never since received	717	18.88
	Not sure	247	6.51
Utilization of the nets last night	Use	2346	61.79
	Did not use	1431	37.69
	Not sure	20	0.52
Presence of unused bed net	Unused old nets	191	22.96
	Unused new nets	433	52.04
Knowledge on benefic of ITN/LLIN	Prevents mosquito bites	445	53.49
	Repels mosquitoes	201	24.16
	Kills mosquitoes	77	9.25
	Kills other insects	103	12.38
	Sleep better	33	3.97
	Protects against malaria	96	11.54
	Protect against other diseases	28	3.37
	Do not know	320	38.46

A total of 734 out of 832, 88.22 % know the malaria disease and regarding on the knowledge on symptoms, the commonest answers were fever (567, 77.25 %), chills (613, 83.5 %) and headache (264, 35.9 %). More than 80 % reported that mosquitoes were responsible for malaria. However, more than 15 % believed that drinking of dirty water (stream water) can cause malaria and 11 % did not know the causes of malaria. Only 305 (36.65 %) said they received the information on malaria within last 1 year. However, 45 of them (14.7 %) unable to mention what information they received. Utilization of the ITNs and knowledge on malaria and information channel of the participants were shown in Tables 3 and 4.

**Table 3:** Utilization of the insecticide treatment bed nets

Indicators	Number	Percent (%)
Proportion of households with at least one ITN	625	75.12
Proportion of households with at least one ITN for every two people	487	58.53
Proportion of population with access to an ITN	3507	75.20
Proportion of existing ITNs used the previous night	1225	70.65

**Table 4:** Knowledge assessment and their information channel

Category	Description	Number	Percent (n = 734)
General knowledge on “malaria”	Know the disease, “malaria”	734	88.22^a^
	Know the blood test for diagnosis	271	36.92
	Know the symptoms of malaria	646	88.01
Cause of malaria	Mosquitoes bite	604	82.29
	Drink/bath of dirty water	177	24.11
	Related to food (e.g., banana)	48	6.54
	Visiting to forest	37	5.04
	Sleeping/staying in the forest	26	3.54
Prevention of malaria	Bed net can prevent “malaria”	403	54.90
	ITN can prevent “malaria”	295	40.19
	Drink of boiled water	65	8.86
	Do not know	115	15.67
Knowledge on anti-malarial	Know any antimalarial	272	29.02
	Artesunate	129	8.31
	Artemether	122	8.04
	Quinine	62	6.95
	Coartem	36	6.95
	Chloroquine	32	6.13
	Others	25	3.41
Source of information	Health staff	213	3.13
	Poster	61	1.77
	Leaflet/Brochures	59	0.95
	TV	51	4.50
	Radio	51	17.30
	Village health volunteer	45	8.58
	Friends/neighbors	25	11.17
	Billboard	23	71.80
	Others	13	37.06
	Do not remember	7	17.57
Received information last time	Within 1 month	33	16.62
	2–6 months	127	8.45
	6–12 months	63	4.90
	Not sure	82	4.36

^a^This percentage was calculated based on all 832 participants and all others were based on the persons who know the disease, malaria (n = 734)

**Fig. 3:**
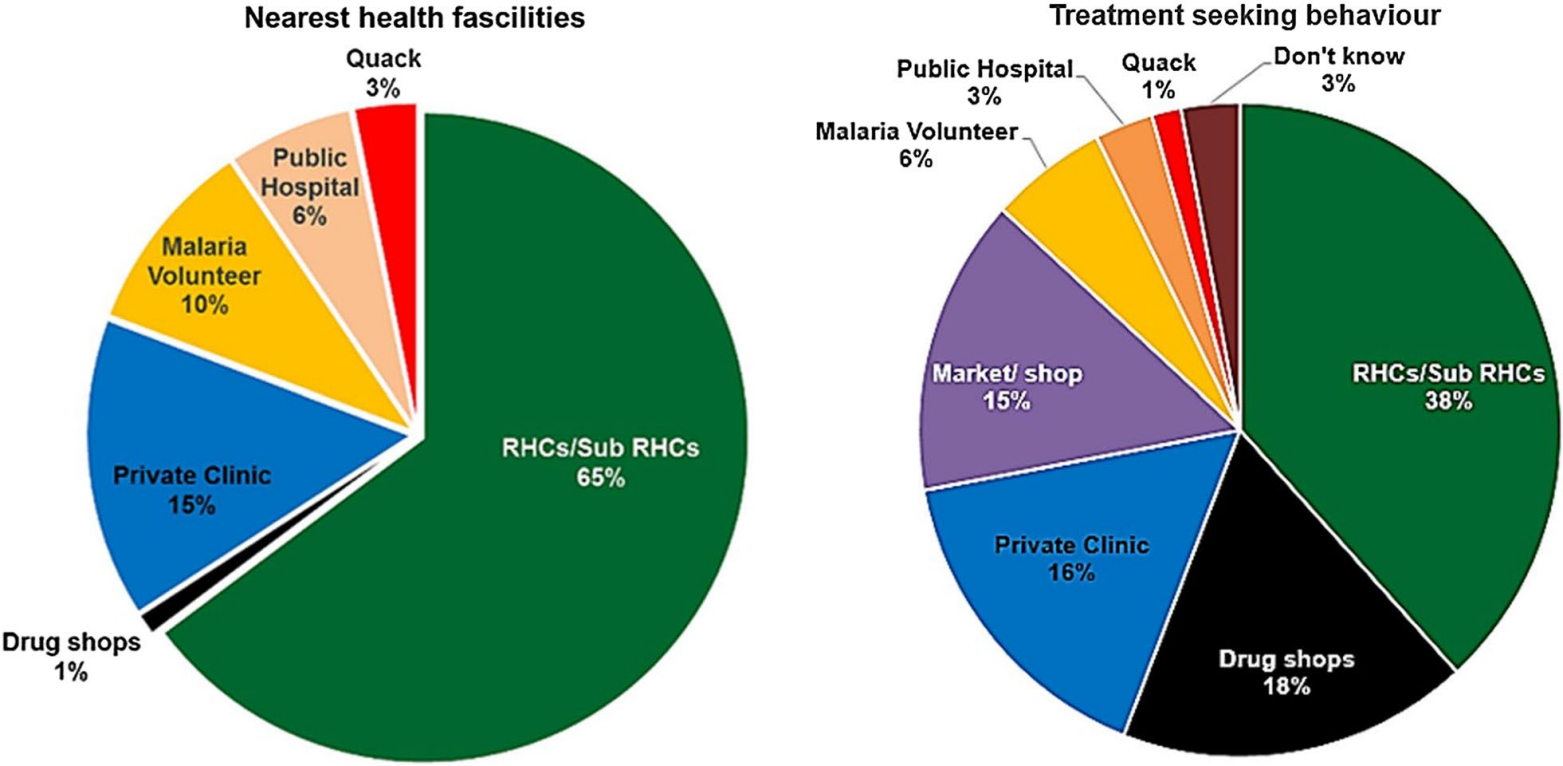
Nearest health facilities vs treatment-seeking behaviour of the participants

In this study, 70 respondents answered that their family had the history of fever within 2 weeks. A total of 83 fever episodes were listed and they were asked for the treatment-seeking pattern. More than 80 % of the fever cases took treatment form outside of the home and their first choice were Rural Health Centre (RHC)/Sub Centre (38.24 %), drug shops (17.65 %) and private clinic (16.18 %), respectively. Nearest health facilities and their treatment-seeking pattern was shown in Fig. 3. Among the 83 fever episodes, only 39 (46.98 %) seek the treatment within 24 h and their choice for treatment was RHC (9, 23 %), private clinic (8, 21 %), drug shop (8, 21 %) and malaria volunteer (3, 8 %).

Based on the retrospective analysis of the fever episodes within last 2 weeks, only 16 out of 83 (19.3 %) episodes took the blood test for detection of malaria (RDT). Among them, only 7 (8.4 % of fever episodes) received the blood test within 24 h. Seven out of 16 blood test become positive by RDT and four cases of them are falciparum infection and relieved by ACT.

Higher education level was found to be relationship with better knowledge on malaria [Odd Ratio (OR) = 3.67], on correct knowledge on cause of malaria (OR = 2.25), on benefit of ITN/LLIN (OR 4.25). Similarly, the persons with correct knowledge on causes of malaria were found to be relationship with utilization of the bed net while sleeping (OR = 4.04). The persons who received information on malaria (BCC exposure), within past 1 year showed the correct knowledge on causes of malaria (OR = 8.55), benefit on LLIN (OR = 9.05) and diagnosis (OR = 9.11). Regarding on the treatment-seeking patterns, the correct knowledge on causes of malaria (OR = 12.11), BCC exposure within 1 year (OR = 8.99) seek the local health care centres and volunteer.

### Qualitative findings

Their risk behaviour and community change mobilization change activities were assessed by focus group discussion and key informal interviews.

One of the township medical officer said:

“BCC activities are believed to be successful. Now most of the people accept to sleep under the bed net and consequently the prevalence of malaria is decreasing”

Another health care provider also mentioned:

“Even some health care personnel do not aware the importance of BCC and we have no routine system for monitoring, supervision and evaluation for BCC”

54 years old women also said as:

“Malaria prevalence have been decreasing because people become better knowledge and awareness on malaria”

However, one of the villagers expressed his belief on causation of malaria as

“Either drinking or bathing in the forest water or cold stream water can cause malaria. It can be prevented by drinking of boiling water only”

Another township medical officer also pointed out the challenges in BCC activities as

“There are many challenges in some villages such as language barriers and security reasons. Some are so reluctant to provide family registration data for bed net distribution”

One of the health staff comment to persuade the high-risk groups as

“BCC activities should be done in target groups who have highest risk for malaria, especially in their free time such as at night”

One malaria volunteer also mentioned as

“Because of the low positive rate and no drug for RDT negative fevers cases, many peoples go to drug shops if they have fever. They don’t want to try the blood test”

One of health providers also said that

“As no one channel for BCC activities is enough in our community, multi-channel approaches based on our resources should be encouraged”.

## Discussion

Behaviour change communication (BCC) are widely considered as a vital tool to use of communication to promote positive health outcomes, based on proven theories and model for behaviour change by interactive processes [12] with communities leading to increase knowledge of malaria instance, stimulating social and communication dialogue, promoting attitude change and demand for information and provided services [13, 14].

In Myanmar, BCC and community mobilization activities have been carrying out as a major component of the MARC programme that include distribution of IEC material such as pamphlets, posters, bill boards, radio talk television short stories series and interpersonal communication including health talks by local health care personals and volunteers [5]. In this study, only 85 (10 %) of the respondents were illiterate and more than half of the household had the media assets, such as television, radio and DVD player, indicating that mass media may be suitable channel for one of the BCC.

RHCs and sub-centres were major nearest health facility for them while a few of them showed the quack and drug sellers as their health care provider. Although every villages had malaria volunteers, they were not nominated as the nearest health care persons.

Ownership as well as utilization of the ITN/LLINs was important in prevention of malaria. Among 832 households, 3797 bed nets were listed and directly observed. Proportion of existing ITNs used in previous night was 70.65 % in this study while proportion of population with access to an ITN was 75.20 %. Nearly 23 % of them had the old nets and 52 % had new unused extra bed nets in their families. It means that in nearly half of the households have the adequate number of the bed nets and free distribution for these families should not be priority.

Most of the ITNs were distributed by local health staff and NGOs, that represented 26 and 10 % of all bed nets indicating the big demand for the all net coverage and retreatment of the all of the nets should be considered. Although overall bed net coverage is just adequate, consideration for replenishment was also important for sustainable coverage and utilization of LLINs.

Nearly 25 % of the all bed nets were re-treated with insecticide and 75 % of the re-treatments were done within 12 months. Interestingly, 1734 (24.9 % of the total ITNs) were retreated. This was also satisfactory and people can get the re-treatment free of charge.

However, 38 % of them unable to mention the benefit of the ITN/LLINs indicating that health education on the treated bed nets should be emphasized. In this study, 88.2 % of the respondent knew the disease “Malaria”. But 11.9 % of them could not mention the sign and symptoms. Only 36.9 % can mention the blood test for malaria diagnosis. More than 80 % correctly answered that bite of mosquitoes cause malaria while a few of them still believe it was caused by bathing or drinking of stream water, cold, eating of unsuitable foods such as banana. These unsatisfactory findings indicated that there were still misunderstanding and beliefs on malaria and it highlighted the information needed to be emphasized in BCC activities.

One of the important findings in this study is only 36.6 % of the participants got the information on malaria within last year and among them, nearly 15 % unable to recognize what information they had received. Health staff were found to be main informer in the study site (69.8 %) followed by poster, brochures, TV and radio and health volunteers. Most of them (>40 %) can mention the drugs “artesunate” and “artemether” as anti-malarials. They also noticed that artesunate was no longer available in the market, but they could still buy the “artemether”. It indicates that correction of the misconception and risk behaviour were still needed in this study population.

Delayed seeking for effective treatment would be a main concern for behaviour barrier to control of malaria [15]. From the 70 households, 83 fever episodes within last 2 weeks were reported. Interestingly, more than 80 % of them took treatment from RHC/sub-centre (38.24 %), drug shops (17.65 %) and private clinic (16.18 %) respectively and only 46.98 % of the fever episodes seek the treatment within 24 h. Although malaria volunteers were assigned in all of villages to get the diagnosis of malaria within 24 h after fever, they were not the first health care provider for most of the fever episodes in this study. This unexpected finding suggests a need to explore the reason for this important issue. As mentioned in qualitative findings, there was no case management guideline and support for RDT negative non-malaria fever cases in low endemic areas and it may have effect on treatment-seeking behaviour of the community.

Our findings highlight the improvement of the behaviour change in the MARC areas comparing with the previous findings [11, 16]. The persons who received the information on malaria had correct knowledge on malaria, LLINs, diagnosis and search the health care personals within 24 h after fever episodes. However, there were many misunderstandings and misconceptions on malaria and risk behaviours in the communities were also noted. It is documented that BCC interventions are most effective when a combination of approaches is used, weaving together mass media, interpersonal communication, advocacy meeting, community participation and structural approaches to promote good behaviours [17, 18].

## Conclusions

Better knowledge about malaria transmission and benefits of using available effective preventive and control measures by the individual households and the community could contribute much to the overall reduction of the malaria burden. Provision of comprehensive behaviour change communication through media/channel that are accessible and appropriate to vulnerable populations would increase the participation of the population in different socioeconomic strata. As the findings of this study highlighted, such efforts should focus on correcting misconceptions about malaria transmission, prevention and universal ITN/LLINs utilization. It is still necessary to emphasize the behaviour change communication and community mobilization by all of the available multi-channel approaches in MARC areas in Myanmar.

### Policy implication and recommendations

- Behaviour change commination and community immobilization activities are still needed to be emphasized.
- Training on BCC in basic health staff and malaria volunteer should be promoted.
- Supervision and monitoring on BCC activities by health staff should be done.
- Since only one BCC activity is not adequate at community level, continuous and powerful activities via multi-channels approaches should be encouraged.
- BCC activities should be emphasized especially on the target groups at convenience time for the audience.
- Operational research on effect of BCC activities should be carried out by using uniform study design in different Tier in MARC before and after the activities.
